# Rice Quality and Yield Prediction Based on Multi-Source Indicators at Different Periods †

**DOI:** 10.3390/plants14030424

**Published:** 2025-02-01

**Authors:** Yufei Hou, Huiyu Bao, Tamanna Islam Rimi, Siyuan Zhang, Bangdong Han, Yizhuo Wang, Ziyang Yu, Jianxin Chen, Hongxiu Gao, Zhenqing Zhao, Qiaorong Wei, Qingshan Chen, Zhongchen Zhang

**Affiliations:** 1 College of Agriculture, Northeast Agricultural University, Harbin 150030, China; 18846132281@163.com (Y.H.); 18348592534@163.com (H.B.); tamannaislam2209@yahoo.com (T.I.R.); wangyizhuo358134@126.com (Y.W.); gaohongxiu_1999@163.com (H.G.); weiqiaorong@neau.edu.cn (Q.W.); qshchen@126.com (Q.C.); 2 National Key Laboratory of Smart Farm Technologies and Systems, Harbin 150030, China; zhaozq@neau.edu.cn; 3 Agricultural Technology Service Center of Beixing Farm, Qitaihe 154625, China; z12031711@163.com; 4 Qixing Branch Agricultural Technology Extension Center, Jiamusi 156300, China; 15246452653@163.com; 5 School of Humanity and Law, Northeastern University, Shenyang 110169, China; ziyangyu@yeah.net; 6 North Oasis Rice Research Institute of Qing’an County, Suihua 152400, China; zhanzhchen@163.com; 7 College of Electrical and Information, Northeast Agricultural University, Harbin 150030, China

**Keywords:** spectral index, growth index, physiological index, univariate linear regression, steowise regression, rice quality, yield

## Abstract

This study aims to develop an effective and reliable method for estimating rice quality indices and yield, addressing the growing need for rapid, non-destructive, and accurate predictions in modern agriculture. Field experiments were conducted in 2018 at the Suiling Water Conservancy Comprehensive Experimental Station (47°27′ N, 127°06′ E), using Longqingdao 3 as the test variety. Measurements included the leaf area index (LAI), chlorophyll content (SPAD), leaf nitrogen content (LNC), and leaf spectral reflectance during the tillering, jointing, and maturity stages. Based on these parameters, spectral indicators were calculated, and univariate linear regression models were developed to predict key rice quality indices. The results demonstrated that the optimal *R*^2^ values for brown rice rate, moisture content, and taste value were 0.866, 0.913, and 0.651, with corresponding RMSE values of 0.122, 0.081, and 1.167. After optimizing the models, the *R*^2^ values for the brown rice rate and taste value improved significantly to 0.95 (RMSE: 0.075) and 0.992 (RMSE: 0.179), respectively. Notably, the spectral index GM2 during the jointing stage achieved the highest accuracy for yield prediction, with an *R*^2^ value of 0.822. These findings confirm that integrating multiple indicators across different growth periods enhances the accuracy of rice quality and yield predictions, offering a robust and intelligent solution for practical agricultural applications.

## 1. Introduction

The growing global population and increasing demand for food have made rice one of the world’s most critical staple crops, with significant implications for both food security and economic stability. As a result, accurately predicting rice quality has become a focal point of agricultural research. Traditional methods for assessing rice quality are time-consuming, typically requiring the rice to be fully harvested before evaluation, making real-time monitoring during the growth period challenging. Therefore, developing rapid, accurate, and non-destructive methods for predicting rice quality during growth is of paramount importance, both theoretically and practically.

Recent advancements in remote sensing technology, particularly the use of spectral indices and unmanned aerial vehicles (UAVs), have revolutionized agricultural monitoring. Spectral data, which provide rich information and clear stratification, are widely employed to diagnose the nutritional status of crops, monitor nutrient content in leaves and grains, and predict crop yield and quality [1]. Chlorophyll indices collected during critical growth stages have been shown to correlate strongly with rice quality metrics such as grain protein content and the percentage of imperfect grains [2]. Similarly, the Normalized Difference Vegetation Index (NDVI) has been widely used for assessing nitrogen uptake and predicting grain yield in rice. Measurements during the panicle differentiation stage showed a positive correlation with total nitrogen uptake, above-ground biomass, and final grain yield, reinforcing its applicability as a predictor of crop performance [3]. Furthermore, multi-temporal NDVI data derived from remotely sensed imagery [4], such as NOAA’s AVHRR, have been successfully utilized to predict rice yield, demonstrating the importance of capturing spectral variability across growth stages [5]. These findings reinforce the utility of spectral indices as a powerful tool for enhancing yield and quality predictions in rice.

In addition to NDVI, other vegetation indices have also shown great promise for crop monitoring. For instance, the evaluation and cross-comparison of spectral indices such as Enhanced Vegetation Index (EVI) and Modified Chlorophyll Absorption in Reflectance Index (MCARI) using Sentinel-2 and WorldView-2 imagery have highlighted the significance of spectral resolution and band selection in improving crop monitoring and prediction accuracy [6]. These advancements provide critical insights into selecting optimal indices for specific crops and growth conditions.

Hyperspectral data, which cover a wide range of wavelengths, have also proven effective in predicting water content in rice seeds, achieving high *R*^2^ values [7]. However, previous studies have been limited to post-harvest assessments, constraining real-time decision-making. This study advances these methods by directly estimating water content using spectral data combined with growth indicators, offering a more convenient and precise approach [8]. Near-infrared spectroscopy (NIRS), another widely used technique, has been applied to develop high-precision models for key rice quality indices such as protein content, amylose content (which affects rice texture), and viscosity (a measure of starch breakdown) [9]. NIRS has also been used to analyze the physicochemical properties of rice, enabling rapid and non-destructive predictions of rice quality [10].

The use of spectral indices for predicting yield has gained significant attention in other crops as well. Spectral reflectance indices have demonstrated utility in predicting wheat yield, revealing their applicability across crop systems [11]. Similarly, remotely sensed spectral indices combined with crop phenology metrics have been effective in forecasting corn yield in Northeast China [12]. Vegetation indices integrated with neural networks have further showcased the potential of advanced machine learning methods for predicting crop yield. Moreover, reviews on the applications of remote sensing in precision agriculture have underscored its role in improving resource management, crop productivity, and sustainability [13]. Such studies highlight the adaptability of spectral indices and remote sensing technology to diverse crops and regions, strengthening their role in precision agriculture.

In rice yield prediction specifically, several models have been developed using environmental variables such as soil temperature, atmospheric temperature, rainfall, and humidity. For instance, a combined principal component analysis and backpropagation neural network model achieved high predictive accuracy for rice yield [14]. Partial least squares regression models have also been employed to integrate vegetation indices across multiple growth stages, demonstrating their reliability for grain yield prediction [15]. Beyond improving prediction accuracy, UAV-based platforms for collecting spectral data have been shown to have significant economic benefits in large-scale farming, such as reducing pesticide application costs and improving efficiency [16]. These findings highlight the growing accuracy, economic viability, and applicability of rice yield prediction models.

Building on these advancements, this study introduces a stepwise regression method that integrates spectral and growth indices across different growth periods to achieve rapid, non-destructive predictions of rice quality and yield. Previous research has shown the effectiveness of individual spectral indices or growth-stage-specific data, but this study advances the field by combining multiple indicators and optimizing predictive models. The results demonstrate improved prediction accuracy, with *R*^2^ values of 0.95 for brown rice rate, 0.913 for water content, and 0.992 for food taste value. Additionally, a simplified single-variable linear model for rice yield prediction achieved an *R*^2^ of 0.822. These findings provide a robust framework for applying spectral and physiological data to precision agriculture, offering practical tools for optimizing rice production.

## 2. Results

### 2.1. The Relationship Between Various Rice Quality Indicators

#### 2.1.1. The Relationship Between Taste Value and Other Rice Quality Indicators

**Figure 1:**
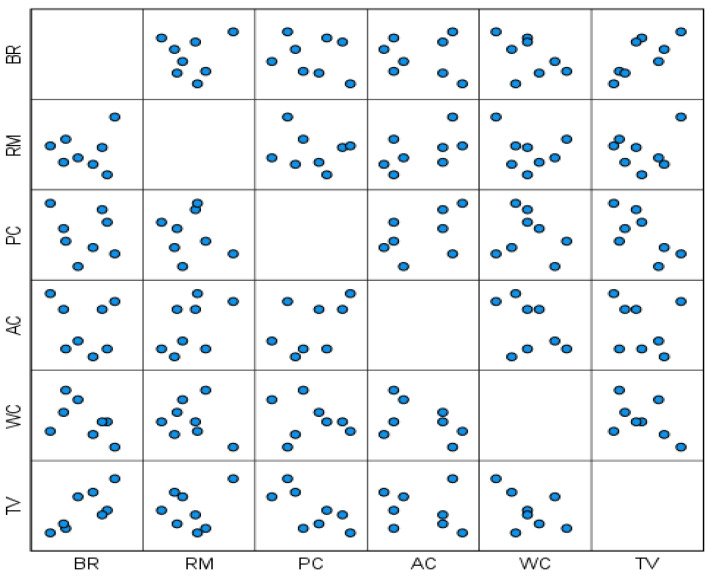
Relationship between rice quality indices.

The food taste value (TV) directly reflects the quality of rice [17] and is influenced by various factors, including protein content (PC), amylose content (AC), and water content (WC). As shown in Figure 1, a significant linear relationship was observed between TV and the other rice quality indicators. To analyze this relationship further, multiple linear regression was performed, with the rice quality indices serving as independent variables and food taste value (TV) as the dependent variable.

#### 2.1.2. Establishing a Multiple Linear Regression Model Using Least Squares Method

To develop a comprehensive prediction model for rice quality, a multiple linear regression model was constructed with food taste value (TV) as the dependent variable and other rice quality indices as independent variables. According to the data analysis in Table 1, the following multiple linear regression equation is obtained to predict the food value (TV).

TV = 110.401 + 1.097 × BR + 0.257 × RM − 8.504 × PC − 2.269 × AC − 3.553 × WC, and the *R*^2^ of the model is 0.908. *p* = 0.013 < 0.05.

The model demonstrated a high goodness of fit, with an *R*^2^ value of 0.908. The *p*-value of 0.013 (<0.05) indicates that the model is statistically significant and passes the F-test. The Variance Inflation Factor (VIF) values were all less than 10, confirming the absence of collinearity among the independent variables. Additionally, the Durbin–Watson (D–W) value of 2.343, close to 2, suggests no autocorrelation between the model’s variables, indicating that the model is well constructed.

### 2.2. Estimation of Rice Quality Indicators Based on Spectral and Growth Indicators at Different Stages

#### 2.2.1. Estimation of Brown Rice Rate Based on Spectral and Growth Indicators at Different Stages

**Figure 2:**
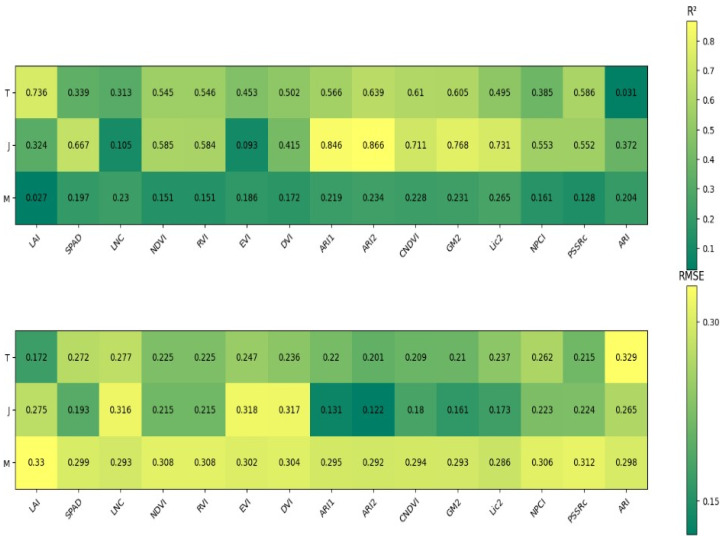
Linear regression accuracy of brown rice rate, spectral indices and growth indices in different periods. T represents tillering stage, J represents jointing stage, M represents maturity stage.

As shown in Figure 2, a single linear regression model was developed to estimate the brown rice rate using spectral and growth indices from different growth stages. The *R*^2^ values for the models ranged from 0.031 to 0.736 at the tillering stage, 0.093 to 0.866 at the jointing stage, and 0.027 to 0.265 at the maturity stage. The model using the spectral index ARI2 at the jointing stage showed the best performance, with an *R*^2^ of 0.866 and an RMSE of 0.122.

#### 2.2.2. Estimate the Moisture Content Based on Spectral and Growth Indicators at Different Stages

**Figure 3:**
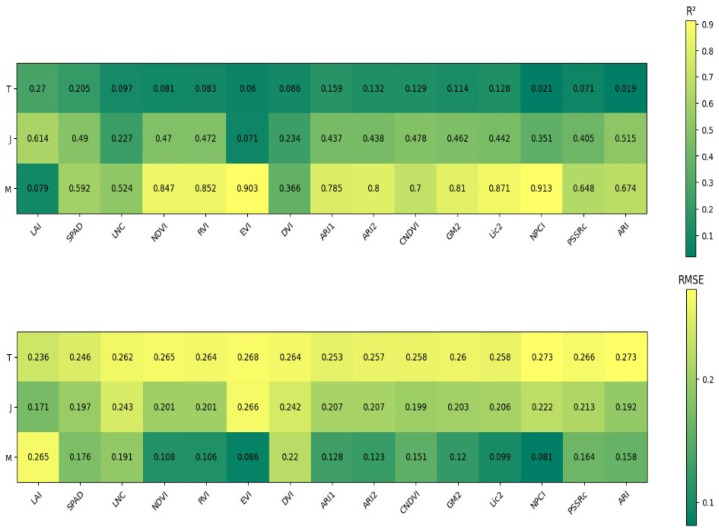
Linear regression accuracy of water content, spectral indices, and growth indices in different periods. T represents the tillering stage, J represents the jointing stage, and M represents the maturity stage.

As shown in Figure 3, a unary linear regression model was developed to estimate water content using spectral and growth indices at different growth stages. The *R*^2^ values for the models ranged from 0.001 to 0.27 at the tillering stage, 0.071 to 0.614 at the jointing stage, and 0.079 to 0.913 at the maturity stage. The model using the spectral index NPCI at the maturity stage performed the best, with an *R*^2^ of 0.913 and an RMSE of 0.081.

#### 2.2.3. Estimate the Taste Value of Different Spectral Indicators and Growth Indicators at Different Stages

**Figure 4:**
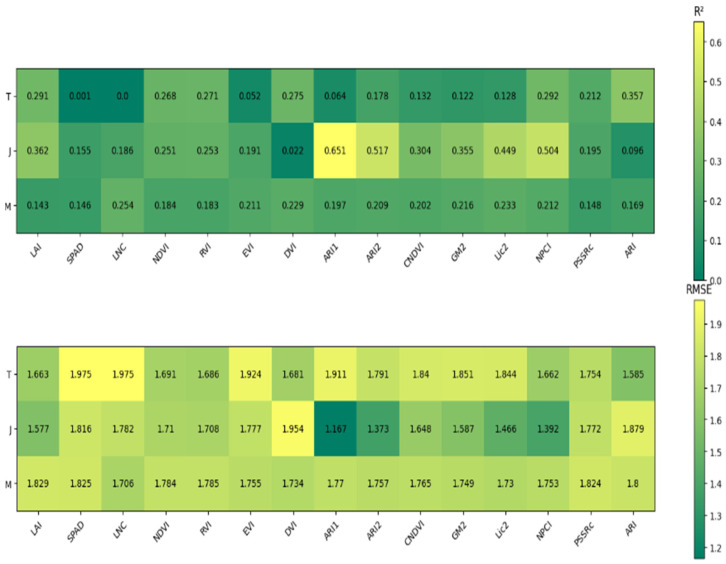
Linear regression accuracy of taste value, spectral indices, and growth indices in different periods. T represents the tillering stage, J represents the jointing stage, and M represents the maturity stage.

As shown in Figure 4, a unary linear regression model was developed to estimate food taste value using spectral and growth indices from different growth stages. The *R*^2^ values for the models ranged from 0 to 0.357 at the tillering stage, 0.022 to 0.651 at the jointing stage, and 0.142 to 0.254 at the maturity stage. The model based on the spectral index ARI1 at the jointing stage performed the best, with an *R*^2^ of 0.651 and an RMSE of 1.167.

#### 2.2.4. Gradually Regress to Establish a Model

To further explore the correlation between the spectral and growth indices at different growth stages and rice quality indicators, correlation analysis was performed. Indicators with a correlation greater than 0.6 with each rice quality index were selected as input variables for stepwise regression analysis. A series of linear regression models were then established to predict rice quality based on stepwise and unary linear regression for each quality index. The results are shown in Table 2.

The stepwise regression analysis revealed that combining the spectral and growth indices at the jointing stage provided the best fit for predicting the brown rice rate and taste value. The coefficient of determination (*R*^2^) for these models was 0.95 for brown rice rate and 0.992 for taste value, with corresponding root mean square errors (RMSEs) of 0.075 and 0.179, respectively. These results demonstrate low model error and high prediction accuracy.

In conclusion, the models established in this study effectively predict brown rice rate, water content, and taste value, offering a reliable tool for rice quality assessment.

### 2.3. Construction of a Rice Yield Estimation Model Based on Spectral and Growth Indicators During the Stagnation Stage

Correlation analysis was conducted between spectral indices, growth indices, and rice yield at the jointing stage. Variables that showed a significant correlation with a yield (*p* < 0.05) were selected as independent variables, including SPAD, LNC, NDVI, RVI, DVI, ARI2, CNDVI, GM2, Lic2, PSSRc, and ARI. Using these variables as independent factors and yield as the dependent variable, a unary linear regression model was developed. The results are presented in Table 3.

The linear regression model based on the spectral index GM2 at the jointing stage provided the best fit for yield prediction, with an *R*^2^ of 0.822 and an RMSE of 572.285.

## 3. Discussion

In this study, we designed an experiment with three nitrogen fertilizer gradient levels and four irrigation treatment levels, theoretically yielding 12 sets of test data. However, due to the cold climate conditions in Northeast China, water pipes in four irrigation groups were frozen and cracked, causing significant water leakage. To maintain the rigor and validity of the results, we excluded data from these four groups—controlled irrigation with low fertilizer, controlled irrigation with medium fertilizer, dry planting with dry pipe low fertilizer, and dry planting with water pipe medium fertilizer. Consequently, our analysis is based on the remaining eight unaffected sets of data. This highlights the importance of accounting for environmental factors and unforeseen challenges in agricultural experiments, which is crucial for ensuring data reliability and applicability [18,19].

We successfully established a multiple linear regression model to predict food taste value (TV) using brown rice percentage (BR), white rice percentage (RM), protein content (PC), amylose content (AC), and water content (WC). This model not only facilitates the estimation of food taste value but also provides insights into the relationships and influence of each quality indicator. Such findings contribute to a scientific basis for improving rice quality through variety selection and gene breeding. These results align with prior studies that integrate physiological and biochemical indicators for predictive modeling, showcasing the potential of such methods for advancing precision agriculture [19,20].

In addition, we constructed linear regression models using spectral and growth indices across different growth periods to predict specific rice quality indicators. The stepwise regression method improved the accuracy of these models, yielding high *R*^2^ values of 0.95 for brown rice rate, 0.913 for water content, and 0.992 for taste value, alongside RMSE values of 0.075, 0.081, and 0.179, respectively. These findings confirm the potential of spectral indices for non-destructive and resource-efficient rice quality assessments. Traditional methods, such as the drying method for moisture content and taste meters for evaluating taste value, are often labor-intensive, time-consuming, and destructive, as highlighted in prior studies [21,22]. Our approach offers a more efficient and sustainable alternative by utilizing spectral indices.

The integration of remote sensing data further strengthens the efficiency and accuracy of predictions, aligning with advancements in precision agriculture that utilize spectral and thermal parameters to monitor crop performance and environmental conditions [19]. Remote sensing technologies not only allow rapid measurement of spectral and physiological indicators but also enable simultaneous predictions of multiple quality metrics. This approach optimizes resource utilization, enhances yield forecasting, and supports informed agricultural management.

Furthermore, we successfully predicted rice yield using spectral indices during the jointing stage, providing early yield estimates that can guide farmers in resource allocation and revenue planning. This aligns with studies emphasizing the importance of early-stage yield predictions for optimizing agricultural practices [23]. However, the current study is limited to specific growth stages, and further integration of spectral data across all growth stages is necessary to enhance the comprehensiveness and accuracy of predictions.

In summary, our findings contribute to advancing precision agriculture by integrating physiological, spectral, and statistical approaches to predict rice quality and yield. Future research should focus on refining experimental designs, integrating dynamic monitoring across all growth stages, and exploring innovative data fusion techniques to overcome environmental constraints and improve predictive models.

## 4. Materials and Methods

### 4.1. Test Materials and Design

The experiment was conducted at the Suiling Water Conservancy Comprehensive Experimental Station in Suihua City, Heilongjiang Province, using the rice variety Longqingdao3, which was transplanted according to a spacing of 30 cm × 10 cm. Three nitrogen fertilizer levels were applied: low (90 kg/hm^2^), medium (120 kg/hm^2^), and high (180 kg/hm^2^). Four irrigation treatments were tested: conventional irrigation, controlled irrigation, dry growing with dry pipe (with no water), and dry growing with water pipe. This resulted in a total of eight experimental groups with varying fertilizer and irrigation combinations: Aa (low fertilizer with conventional irrigation), Ab (medium fertilizer with conventional irrigation), Ac (high fertilizer with conventional irrigation), Bc (high fertilizer with controlled irrigation), Cb (medium fertilizer with dry growing and dry pipe), Cc (high fertilizer with dry growing and dry pipe), Da (low fertilizer with dry growing and water pipe), and Dc (high fertilizer with dry growing and water pipe). The experimental design followed a randomized block layout with 24 plots, each replicated twice. Fertilizer was applied in a nitrogen, phosphorus, and potassium ratio of 2:1:2, with base tiller and ear fertilizers applied at a 5:2:3 ratio.

Conventional irrigation was managed according to local agricultural practices, with the field drained during the late tillering period and a shallow water layer maintained during the other growth stages, drying naturally at the yellow ripening stage. The controlled irrigation treatment was managed similarly, while dry growing treatments involved no irrigation throughout the entire growth period, except for the dry water pipe treatment, which included a water layer established after the four-leaf stage.

### 4.2. Measurement Items and Methods

The primary rice quality indices measured in the study included brown rice rate (BR), rice milling rate (RM), protein content (PC), amylose content (AC), water content (WC), and taste value (TV). These parameters were assessed using standard procedures to evaluate the overall quality of the rice under different fertilizer and irrigation conditions.

#### 4.2.1. Determination of Rice Quality Indices

- Brown Rice Rate and Polished Rice Rate: Samples of 500 g were collected from the harvested rice in each treatment, with three repetitions per treatment. The brown rice yield was determined using an LMG rice milling machine (brown rice weight/rice weight × 100%). Twenty grams of brown rice (in triplicate) were milled using a small white mill to produce fine rice, and the milled rice rate was calculated as (milled rice weight/rice weight × 100%) [24].
- Protein Content, Amylose Content, Moisture Content, and Taste Value: Polished rice samples were analyzed using a PS-500 taste analyzer (Shizuoka Machinery Co., Ltd., Shizuoka, Japan) to determine protein content, amylose content, moisture content, and taste value.

#### 4.2.2. Criteria for Selecting Spectral and Physiological Indices

The selection of spectral indices was based on their established relevance to crop health, nutrient status, and yield prediction in previous studies. Indices such as NDVI and GM2 were chosen for their ability to monitor vegetation health and nitrogen content across growth stages, while ARI1 and ARI2 were selected for their sensitivity to chlorophyll content and stress detection. Physiological indices, including SPAD, LAI, and LNC, were chosen for their proven correlation with crop health, chlorophyll content, and nitrogen status, which are critical for yield and quality estimation. These indices were prioritized for their ease of measurement, non-destructive nature, and strong predictive performance in prior research [25].

#### 4.2.3. Spectral Data Collection and Calibration

Spectral reflectance data of rice leaves were measured using an SVC HR768i spectroradiometer (American SVC), (Spectra Vista Corporation, Poughkeepsie, NY, USA), with a spectral detection range of 350–2500 nm and spectral resolutions of 3.5, 8.5, and 6.5 nm. A handheld blade spectral detector with an integrated halogen light source ensured consistent illumination. The spectrometer was calibrated before each session using a standard white reference panel to eliminate ambient light interference and maintain measurement accuracy. Dark current correction was conducted by covering the sensor to record baseline noise, which was subtracted from all measurements.

For each treatment level at the tillering, jointing, and maturity stages, three representative rice plants were selected, and six groups of leaves at the top canopy were measured per plant. Reflectance data for each leaf were collected using a leaf clip attachment to ensure consistent positioning and minimal external interference. Three spectral readings were recorded per leaf, and the average reflectance was calculated to minimize variability. Preprocessing included the nine-point weighting method for noise reduction, resampling at 1 nm intervals using ENVI 5.1, and continuum removal to normalize the reflectance data to a range of 0–1, highlighting absorption and reflection features in the spectral curve. These steps ensured reliable and accurate spectral data for analysis [25].

#### 4.2.4. Measurement of Physiological Indices

- Leaf Area Index (LAI): Measured using an LAI-2200C canopy analyzer (LI-COR Biosciences, Lincoln, NE, USA) to evaluate canopy structure and light interception.
- Chlorophyll Content (SPAD) and Leaf Nitrogen Content (LNC): Measured non-destructively using a TYS-4N plant nutrition tester (Zhejiang TOP Cloud-Agri Technology Co., Ltd., Hangzhou, China). Three measurements per leaf were averaged to obtain the final SPAD and LNC values. SPAD values were collected based on the optical concentration difference between 650 and 940 nm, ensuring high repeatability and accuracy.

#### 4.2.5. Process of Statistical Standardization

To ensure consistency and comparability across spectral and physiological indices, statistical standardization was applied. All raw data were normalized using Z-score normalization, which standardizes variables to a zero mean and unit variance. This eliminated scale differences among indices and improved the robustness of regression models. Spectral indices were further smoothened and standardized during preprocessing to minimize noise and variability, enhancing their predictive reliability.

#### 4.2.6. Yield Determination

At maturity, rice plants from the central 1 m × 1 m area of each plot were harvested to determine the actual yield. Yield data were combined with standardized spectral and physiological indices to develop prediction models.

### 4.3. Data Analysis

#### 4.3.1. Rice Quality Index Data Preprocessing

Outliers in the dataset were identified using boxplot analysis and corrected using the mean-value correction method to ensure consistency and accuracy. Post-outlier correction, all analyses were conducted on the cleaned dataset.

#### 4.3.2. Spectral Data Preprocessing

Spectral data collected using the SVC HR768i spectroradiometer (Spectra Vista Corporation, Poughkeepsie, NY, USA) were smoothened using the SVC HR-768 PC software (version 1.13). The nine-point weighting method was applied to reduce noise, while inflection points at various sampling intervals were corrected to improve data quality. Data were resampled at 1 nm intervals, focusing on the spectral range of 440–2400 nm. The resampled data were normalized using continuum removal to highlight absorption and reflection features in the spectral curves. This process standardized the spectral data for subsequent modeling.

#### 4.3.3. Selection of Spectral Vegetation Indices

Based on extensive literature and the spectral features derived from multispectral data, 12 vegetation indices were selected for analysis (Table 4). These indices were chosen based on their relevance in previous studies and their ability to reflect vegetation health, chlorophyll content, and other physiological characteristics.

#### 4.3.4. Statistical Models

- Simple Linear Regression Model:

A linear relationship was modeled between individual vegetation indices (e.g., NDVI and EVI) and specific quality metrics, such as protein content and water content. The general equation is as follows:

$$y=\beta_{0}+\beta_{1}x+\epsilon$$

where *y* represents the dependent variable (e.g., rice quality metric), *x* represents the independent variable (vegetation index), $\beta_{0}$ is the intercept, $\beta_{1}$ is the slope coefficient, and ϵ is the error term.

- Multiple Linear Regression Model:

This model incorporated multiple independent variables to predict a single dependent variable. It is expressed as:

$$y=\beta_{0}+\beta_{1}x_{1}+\beta_{2}x_{2}+\cdots +\beta_{n}x_{n}+\epsilon$$

where $x_{1},\;x_{2}$, …, $x_{n}$ are the independent variables (e.g., multiple vegetation indices or physiological parameters).

Stepwise Multiple Regression:

Stepwise regression was employed to optimize variable selection and improve model accuracy. Three methods were used:

- Forward Selection: Iteratively adds variables that significantly improve the model fit.
- Backward Elimination: Starts with all candidate variables and iteratively removes non-significant ones.
- Stepwise Regression: Combines forward selection and backward elimination, retaining only variables that meet the statistical significance criteria at each step.

#### 4.3.5. Data Processing

Data organization and preliminary analyses were performed using Excel 2021, while advanced statistical modeling was conducted using SPSS 27.0. Model accuracy was assessed using the coefficient of determination (*R*^2^) and root mean square error (RMSE). These metrics ensured robust evaluation of model performance in predicting rice quality and yield.

## 5. Conclusions

This study developed multiple linear regression models using food taste value (TV) as the dependent variable and brown rice rate (BR), milled rice rate (RM), protein content (PC), amylose content (AC), and water content (WC) as independent variables. The results demonstrated that BR and RM were positively correlated with TV, while PC, AC, and WC showed negative correlations, with PC being the most influential factor. These findings emphasize the potential for adjusting PC and WC during rice breeding and processing to improve eating quality.

High-precision models were also established to predict rice quality indices, achieving *R*^2^ values of 0.95, 0.913, and 0.992 for brown rice rate, water content, and taste value, respectively, with corresponding RMSE values of 0.075, 0.081, and 0.179. These models offer practical tools for non-destructive, rapid, and accurate quality assessments. Additionally, the unary regression model utilizing the GM2 spectral index at the jointing stage demonstrated the highest accuracy for yield prediction (*R*^2^ = 0.822, RMSE = 572.285), enabling early yield estimation.

To implement these models in real-world scenarios, they could be integrated into precision agriculture workflows, leveraging UAVs or handheld spectral sensors for data collection. This would allow farmers and agronomists to monitor rice quality and yield dynamically, optimizing resource allocation, irrigation strategies, and fertilization practices. However, operational challenges such as calibration requirements for equipment, data processing complexity, and environmental variability should be addressed to maximize model applicability and reliability. Future research should focus on validating these models across diverse environmental conditions and exploring their scalability for large-scale agricultural systems.

## Figures and Tables

**Table 1:** Regression coefficient matrix, collinearity test, and F-test for predicting taste value of rice quality indices.

	*B*	Standard Error	VIF	Tolerance
constant	110.401	171.282	-	-
BR	1.097	1.495	2.596	0.385
RM	0.257	0.674	3.644	0.274
PC	−8.504	3.620	4.084	0.245
AC	−2.269	4.076	3.109	0.322
WC	−3.553	1.996	1.885	0.530
*R* ^2^			0.908	
F			*F*(5,5) = 9.851, =0.013*p*	
D–W value			2.343	

**Table 2:** Linear regression model of spectral indices, growth indices and rice quality indicators.

Quality Indicators	Reproductive Period	Factors	Regression Equations	*R* ^2^	RMSE
BR	Jointing stage	ARI, ARI2	BR = 76.580 − 0.615 * ARI − 5.343 * ARI2	0.95	0.075
WC	Maturity stage	NPCI	WC = 9.767 − 6.865 * NPCI	0.913	0.081
TV	Jointing stage	ARI1, CNDVI, LAI	TV = 58.233 − 2598.633 * ARI1 + 1.775 * LAI − 61.226 * CNDVI	0.992	0.179

**Table 3:** Unitary linear regression models for predicting rice yield.

Factors	Regression Equations	*R* ^2^	RMSE
SPAD	Y = −4651.164 + 337.417 * SPAD	0.705	738.944
LNC	Y = 5462.578 + 134.886 * LNC	0.189	1222.2
NDVI	Y = −87,357.412 + 118,579.896 * NDVI	0.771	649.408
RVI	Y = 28,840.137 – 191,939.879 * RVI	0.770	650.984
ARI2	Y = 109.777 – 14,471.137 * ARI2	0.822	573.145
CNDVI	Y = −1416.363 + 23,220.135 * CNDVI	0.798	609.743
GM2	Y = −2212.498 + 3501.826 * GM2	0.822	572.285
Lic2	Y = −5808.406 + 16,089.978 * Lic2	0.705	737.184
PSSRc	Y = −14,921.764 + 2900.088 * PSSRc	0.805	599.528
ARI	Y = 20,865.025 + 3374.634 * ARI	0.403	971.355

**Table 4:** Spectral vegetation indices and calculation formulas.

Indicator Abbreviation	Computing Formula
NDVI	(R800 − R680)/(R800 + R680) [26]
RVI	R680/R800 [27]
EVI	2.5 * (R800 − R680)/(R800 + 6 * R680 − 7.5 * R450 + 1) [28]
DVI	R800 − R680
ARI1	(1/R550) − (1/R700) [29]
ARI2	R800 * ARI1 [29]
CNDVI	(R790 − R550)/(R790 + R550) [30]
GM2	R750/R700 [31]
Lic2	R440/R690 [32]
NPCI	(R680 − R430)/(R680 + R430) [33]
PSSRc	R800/R470 [34]
ARI	R700 − R550

Rλ is the spectral reflectance at the λ wavelength.

## Data Availability

No new data were created or analyzed in this study. Data sharing is not applicable to this article.
